# Plant HKT Channels: An Updated View on Structure, Function and Gene Regulation

**DOI:** 10.3390/ijms22041892

**Published:** 2021-02-14

**Authors:** Janin Riedelsberger, Julia K. Miller, Braulio Valdebenito-Maturana, Miguel A. Piñeros, Wendy González, Ingo Dreyer

**Affiliations:** 1 Centro de Bioinformática, Simulación y Modelado (CBSM), Facultad de Ingeniería, Campus Talca, Universidad de Talca, 1 Poniente No. 1141, Casilla 721, 3460000 Talca, Chile; braulio.valdebenito@utalca.cl (B.V.-M.); wgonzalez@utalca.cl (W.G.); idreyer@utalca.cl (I.D.); 2 Plant Biology Section, School of Integrative Plant Science, Cornell University, Ithaca, NY 14853, USA; jkm239@cornell.edu (J.K.M.); map25@cornell.edu (M.A.P.); 3 Robert W. Holley Center for Agriculture and Health, USDA-ARS, Cornell University, Ithaca, NY 14853, USA; 4 Millennium Nucleus of Ion Channels-Associated Diseases (MiNICAD), Universidad de Talca, 3460000 Talca, Chile

**Keywords:** HKT channels, sodium transport, potassium transport, selectivity filter, gene regulation

## Abstract

HKT channels are a plant protein family involved in sodium (Na^+^) and potassium (K^+^) uptake and Na^+^-K^+^ homeostasis. Some HKTs underlie salt tolerance responses in plants, while others provide a mechanism to cope with short-term K^+^ shortage by allowing increased Na^+^ uptake under K^+^ starvation conditions. HKT channels present a functionally versatile family divided into two classes, mainly based on a sequence polymorphism found in the sequences underlying the selectivity filter of the first pore loop. Physiologically, most class I members function as sodium uniporters, and class II members as Na^+^/K^+^ symporters. Nevertheless, even within these two classes, there is a high functional diversity that, to date, cannot be explained at the molecular level. The high complexity is also reflected at the regulatory level. HKT expression is modulated at the level of transcription, translation, and functionality of the protein. Here, we summarize and discuss the structure and conservation of the HKT channel family from algae to angiosperms. We also outline the latest findings on gene expression and the regulation of HKT channels.

## 1. Introduction

More than 25 years ago, in 1994, the first HKT channel was cloned from wheat roots—*TaHKT2;1* [1]. HKT proteins were initially characterized as high-affinity K^+^ (potassium) transporters, leading to the HKT acronym. It was soon demonstrated that HKT channels could transport other cations, namely sodium (Na^+^) ([2],for concise synopsis see: [3]).

HKTs are part of the Trk/Ktr/HKT family and share a characteristic structure made up of four transmembrane domain-pore domain-transmembrane domain units (MPM_1_-MPM_4_) (Figure S1). In phylogenetic analyses, the HKT family splits into two subgroups: class I and class II [4,5,6]. The selectivity filter sequence and physiological features generally define class assignment. Four glycine residues distributed over the pore loops form the selectivity filter. Classes I and II are differentiated by the presence of either a serine residue (SGGG) or a glycine residue (GGGG) in the first pore loop, which mostly translates into class-specific differences in ion conduction [7]. However, exceptions to this classification have been found, questioning the classification’s accuracy [5,8,9,10,11].

Generally, class I HKTs (SGGG) are characterized as Na^+^ uniporters, while class II HKTs (GGGG) mediate the Na^+^ and K^+^ symport. Class II proteins are also affected by the external ion composition, which was repeatedly shown to modulate ion conduction. This mutual effect was described for the interplay of Na^+^ and K^+^ ions affecting the transport of the other cation, as well as for cations like lithium, magnesium, or calcium modulating Na^+^ or K^+^ transport [12,13,14,15,16,17,18,19,20]. The extracellular ionic environment also regulates gene expression, such that K^+^ shortage often leads to upregulation of *HKT* expression, while rising K^+^ concentrations result in downregulation [15,21,22]. Previous studies have indicated that high Na^+^ concentration also results in downregulation of *HKT* expression. Sunarpi et al. demonstrated that *AtHKT1;1* is upregulated upon increasing Na^+^ concentrations, but only up to 30 mM. This effect is revoked at Na^+^ concentrations above 30 mM and is completely abolished at 75 mM [23].

Physiologically, HKT channels play roles in the plant Na^+^-K^+^ homeostasis and Na^+^ and K^+^ uptake (reviewed by [8,24,25]). Na^+^ and K^+^ transport within the plant is carried out by class I HKTs and is linked to salt tolerance [7,23,26,27,28,29,30,31]. Class II HKTs facilitate Na^+^ and K^+^ uptake, which is especially beneficial under K^+^ starvation conditions [12,16,19,32,33,34,35]. The Na^+^-K^+^ symport ability is advantageous under conditions of K^+^ starvation due to the capacity of Na^+^ to functionally replace K^+^ to a certain extent, bridging the short-term lack of K^+^ and ensuring the survival of the plant [36]. This is in line with the previously described upregulation of HKT transcripts under K^+^ shortage. Also, the conduction behavior is affected by the prevailing extracellular cation concentration. It was shown that, for example, Na^+^ currents through OsHKT2;2 are enhanced with low K^+^ in the background and reduced with increasing K^+^ concentrations [12,14]. This observation complies with Na^+^ being only a temporary K^+^ replacement, and as soon as K^+^ is available, the Na^+^/K^+^ ratio has to be restored.

Recent reviews summarized Na^+^ transport systems in glycophytes and halophytes [37,38]. In this review, we focus on examining HKT expression and structural features associated with their functionality, specifically their selectivity. For this purpose, we first revise the primary structure of HKT channel sequences from algae to angiosperms and discuss known and new sequence features in the context of their evolution. In the second part, we summarize recent findings regarding HKT-mediated transport regulation at the gene expression level.

## 2. HKT Sequence and Structure

To review and consolidate the vast existing functional and structural information on the HKT family, we will first explore protein similarity and divergence of HKT family members from an evolutionary perspective. This examination allows us to revise the association between the degree of conservation in structural motifs associated with substrate selectivity and recognition, with the functional plasticity observed in HKT-mediated ion transport.

### 2.1. Evolution of the HKT Family

**Figure 1:**
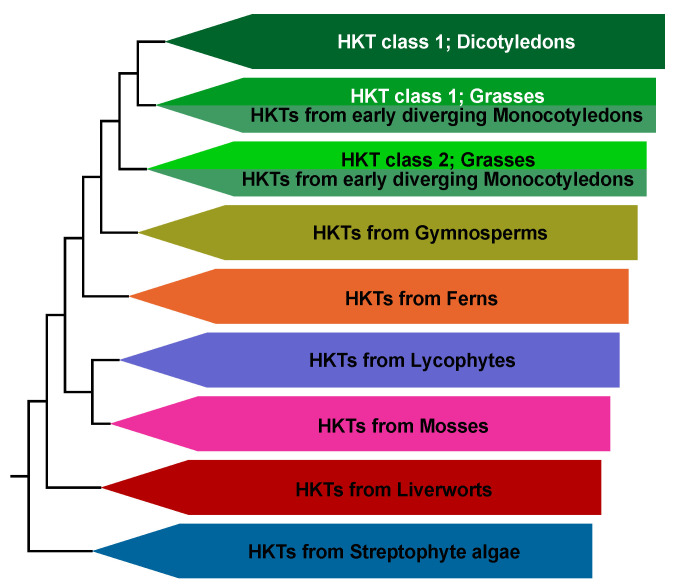
Condensed phylogeny of plant HKT channels. Evolutionary history derived from the phylogenetic tree shown in Figure S1. While there was mostly a gradual evolution of HKTs until the occurrence of seed plants, in monocotyledon angiosperms they split into two sub-branches. Class 2 HKTs can only be found in monocotyledons, while HKTs of class 1 can be found in monocotyledons and dicotyledons.

HKT channel sequences have been identified in plant species from algae to angiosperms. While angiosperm HKTs are well-studied, experimental data of evolutionarily older HKTs is scarce [5]. Figure 1 illustrates the phylogenetic relationship of HKT channels from nonflowering plants, gymnosperms, and angiosperms classified according to the species tree of The Plant Genomics Resource Phytozome 12 [39]. The evolution of HKT proteins was mostly linear in non-seed-making plants until the family split into two subgroups in seed-making plants—class I and class II HKTs [6]. It has been hypothesized that class I and II *HKT* genes originated through gene duplication and divergence, and evolved separately [40]. Monocotyledons have representatives of both classes, while in dicotyledons, only class I HKTs have been identified, and class II has not been established (Figure 1 and Figure S1). This may suggest that class II evolved exclusively in monocotyledons. Nevertheless, based on the currently available data, it cannot be conclusively ruled out that the separation of the two classes occurred before the separation of mono- and dicotyledons with a subsequent loss of class II in dicotyledons. Interestingly, class II members seem closer, sequence-wise, to non-seed-making plant members, while class I HKTs show new sequence features (see below). In the following, we will use the HKT phylogeny and the underlying sequence and structural features to discuss published functional data and place it in a broader context. This comparison manifests intriguing insights into the history of nonflowering plants, gymnosperms, and angiosperms.

### 2.2. Pore Domains Bear a High Degree of Conservation

**Figure 2:**
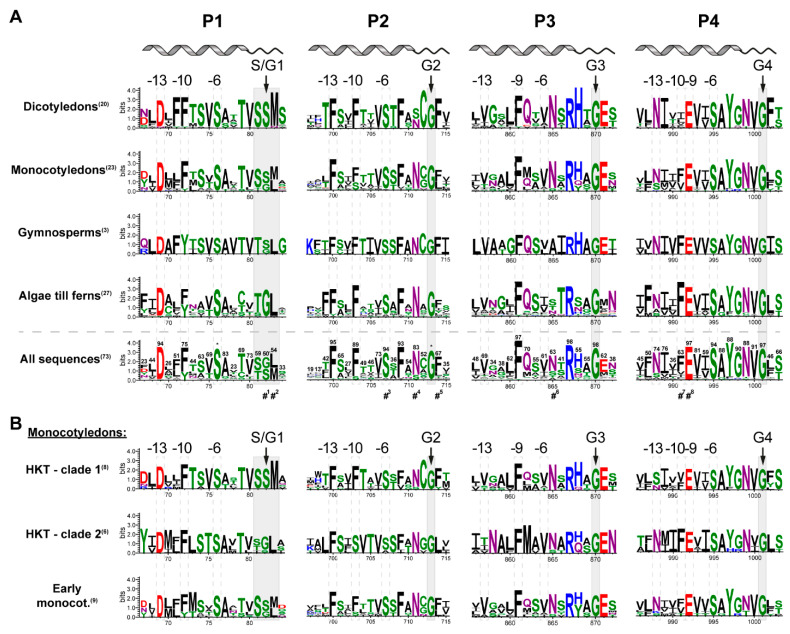
Motifs and conserved amino acids in HKT channel pore regions. Sequence logos based on 73 sequences used for the multiple sequence alignment in Figure S2 and the phylogenetic tree in Figure S1 were generated by WebLogo 3.7.4 [42,43]. (**A**) Logos of the four pore domains P1–P4 for sequences of algae to ferns, gymnosperms, monocotyledons, dicotyledons, and all 73 sequences together. The illustration above the sequence logos represents an approximation of the secondary structure of each pore domain, indicating the pore helix and pore loop. Numbers in parenthesis next to the names of evolutionary groups indicate the number of sequences used for the generation of the logos. Please note that the sequence logo for gymnosperms was generated with only three sequences. For the sequence logos based on all sequences, the percentage of the most frequent amino acid per position is indicated. Numbers above amino acids are percentages. The asterisk indicates residues with 100% presence in all structures. A quotation mark (”) indicates that two amino acids appeared with the highest frequency in the alignment (e.g., 50” indicates that the two most frequent amino acids each appear in 50% of the sequences). S/G1, G2, G3, and G4 indicate the selectivity filter residues. A motif switch in P1, including the selectivity filter residue, is shaded, as well as the selectivity filter residues in P2 to P4. Dashed boxes mark amino acids six, nine or ten, and thirteen positions upstream of selectivity filter residues. Residues are coloured according to their chemical properties: polar residues (G, S, T, Y, C) in green, neutral residues (Q, N) in purple, basic residues (K, R, H) in blue, acidic residues (D, E) in red, and hydrophobic residues (A, V, L, I, P, W, F, M) in black. The number signs below the logos indicate positions for which mutations are available. Mutations are described in the following references: #1 [7], #2 [7], #3 [2], #4 [44], #5 [2], #6 [45], #7 [41], #8 [41]. (**B**) Sequence logos of the four pore regions specified for early angiosperms, class I, and class II HKTs. Highlights as in A.

Several structural features of HKT channels have been studied so far. These include conserved key residues as parts of the selectivity filter and pore domains, residues in the second transmembrane domain of unit 4 (U4M2), the intracellular loop between units 1 and 2, as well as an extracellular cation coordination site (for summary, see: [9,14,24])). Nevertheless, the HKT family is, functionally speaking, exceptionally diverse, and key functional differences are not yet understood at the molecular level. Therefore, the primary sequences of HKTs hold additional peculiarities worth investigating. Figure 2 illustrates a high degree of amino acid sequence conservation in the four pore domains of the distinct evolutionary groups. The pore domain of unit 4 (P4) appears to be the most conserved over time. This observation has also been highlighted by Diatloff and colleagues while comparing HKT’s primary sequence with yeast Trk sequences [41]. Nine residues are conserved in more than 80% of HKT sequences examined, defining the sequence motif EVxSAYGNVG (Figure 2A, All sequences, P4, Figure S2). Some deviations from this P4 motif are evident in monocotyledons and non-seed-making plant sequences (algae to ferns). In contrast to P4, the first three pore domains appear less conserved over time when all sequences are taken into account. P1 contains three residues—D[…]SA—P2 has six residues—FxxFxxxSxFxNxG—and P3 has three residues—F[…]RxxG—that are conserved in more than 80% of sequences. Additionally, monocotyledons, together with non-seed-making plants, show a higher sequence variety than other groups. 

However, defined positions relative to the selectivity filter residues are often highly conserved. Amino acids upstream of the selectivity filter at position –6, –9/–10, and, in part, –13 are conserved (dashed boxes in Figure 2). In three of the four units, a serine residue is highly conserved at the sixth position upstream of the selectivity filter residues. It is only in P3 that this position is not conserved, and, at least in seed-making plants, it is predominantly conserved as a valine residue. However, in angiosperms, the neighboring position –5 is conserved as an asparagine (N).

Similarly, the residues at the 9th (P3 and P4) or 10th (P1 and P2) position upstream of the selectivity filter are conserved as phenylalanine (F) throughout the first three pore domains and as a glutamate (E) in the 4th pore domain. Interestingly, in the P4 position –10 holds a conserved phenylalanine (F) in non-seed-making plants, which disappears in dicotyledon angiosperms but is conserved in clade II monocotyledons (Figure 2B). Position –13 upstream of the selectivity filter is highly conserved in P1 and P2. In P3, this position is somewhat conserved as a valine (V), similar to position –6 in P3. In P4, position –13 is mostly conserved as an asparagine (N), except in clade I monocotyledons, early angiosperms, and non-seed-making plants.

Substitutions of some of the mentioned residues resulted in alterations of the conduction behavior. For example, expressing wild-type TaHKT2;1 in yeast produced Na^+^ toxicity and resulted in growth inhibition [2]. Using this yeast screening system, two TaHKT2;1 mutants that revoked the sensitivity of the yeast towards high salinity conditions were identified. One was TaHKT2;1-A240V; residue A240 corresponds to the sixth position upstream of the selectivity filter glycine in P2. Exceptionally, TaHKT2;1 and HcHKT2;1 contain an alanine at this position, instead of the otherwise conserved serine. Substitution of alanine by valine at this position (i.e., A240V) altered the functional characteristics of TaHKT2;1 [2]. As described for the class II HKT channels, TaHKT2;1 transports Na^+^ and K^+^ ions. The A240V mutation resulted in reduced Na^+^ transport and increased K^+^ uptake. Mutation of the position 5 upstream of the selectivity filter residue in P3 (TaHKT2;1-N365S) reduced the Na^+^ transport at low and high Na^+^ concentrations, also indicating the importance of this position for ion conduction [45]. Mutations of residues at the 9 (TaHKT2;1-E464Q) and 10 (TaHKT2;1-F463L) positions upstream of the selectivity filter in P4 were identified based on sequence analysis and characterized in yeast uptake experiments. The highly conserved glutamate (E) at position -9 in P4 and the phenylalanine (F) at position –10, which is conserved in clade II monocotyledons and evolutionary older HKTs, both resulted in reduced Na^+^ transport upon mutation. TaHKT2;1-F463L additionally showed a reduced affinity for K^+^ transport [41].

**Figure 3:**
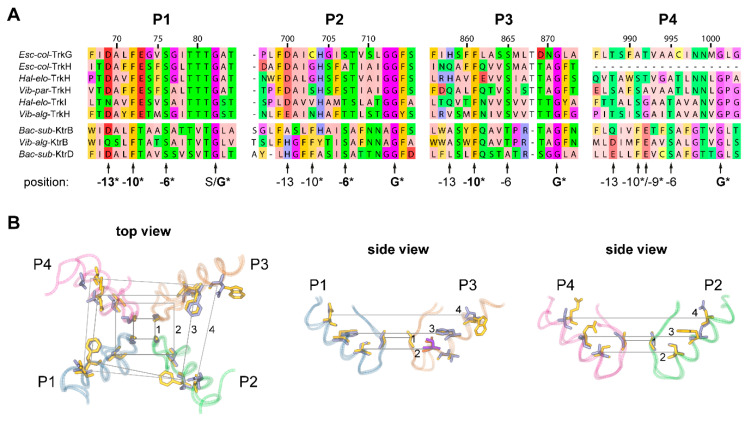
Conserved residues in the pore helix may stabilize the pore loops. (**A**) Sequence alignment of the pore domains P1 to P4 of Trk and Ktr. Positions above the alignment coincide with the numbering in Figure 2 for easier comparison. Selectivity filter residues and residues 6, 9/10, and 13 positions upstream of the selectivity filter are marked. The asterisk and a bold format indicate conservation of the residue in Trk and Ktr proteins, while the asterisk in normal font indicates conservation in only one of the groups Ktr or Trk. The alignment is coloured based on physicochemical properties according to the Zappo colour scheme of Jalview. (**B**) Superposition of the pore domains of KtrB (PDB ID 4J7C) and TrkH (PDB ID 6V4L) crystal structures. The protein backbone is displayed as tubes with each pore domain in a different colour. Selectivity filter residues (plane 1) and residues 6 (plane 2), 10 (plane 3), and 13 (plane 4) positions upstream are presented in licorice. Residues from TrkH are blue, those from KtrB yellow. The side view of P3 additionally illustrates the orientation of the residue 5 positions upstream of the selectivity filter in orange (KtrB) and purple (TrkH).

Altogether, these mutations demonstrate that the mentioned conserved positions are key determinants for ion conduction characteristics. Structurally, in the three-dimensional space, these residues are located in the pore helix, which is positioned in the back of the pore loop that lines the conduction pathway and contains the selectivity filter residues. Figure 3 illustrates their position relative to the selectivity filter residues. Most of the residues are oriented towards the pore loop, quite likely serving a stabilizing function. Interestingly, the residue at position –6 relative to the selectivity filter in P3 (valine (V) in plant HKTs) is oriented away from the filter. However, the neighboring residue at position –5 (conserved asparagine (N) in plant HKTs), which resulted in altered Na^+^ affinity upon substitution, is instead oriented towards the pore loop (Figure 3B, orange and purple residues). Structural data are based on the crystal structures of KtrB from *Bacillus subtilis* and TrkH from *Vibrio parahaemolyticus*. Mutating residues behind the pore loop may directly affect the shape of the pore region above the selectivity filter, thereby impacting the transport characteristics of HKT channels, as was demonstrated by the above-mentioned mutations. It will be interesting to see whether mutations of the potentially stabilizing residues allow the alteration of the HKT’s selectivity and conduction characteristics, and whether it can be used to modulate HKT functionally and exploit this feature to alter and improve Na^+^ and K^+^ homeostasis in plants. Also compelling to research is the reason for the highly conserved fourth pore domain, which has been shown to play a role in ion transport but comprises a higher level of conservation than other pore domains.

### 2.3. A Selectivity Filter Motif in the First Pore Region Changed

It has been established that the nature of the residue of the first pore loop forming the selectivity filter determines the cation selectivity of HKT channels [7,46]. Generally, a serine residue is associated with Na^+^-selective channels, while a glycine substitution at this position results in permeation of multiple cations. Residues in the P2 to P4 region of the selectivity filter are highly conserved throughout all evolutionary stages (G in P2: 100%, P3: 98%, P4: 97%, gray boxes in Figure 2A). In contrast, the serine/glycine substrate specificity determined by P1 at the selectivity filter experienced a change, where the Na^+^-selective configuration (SGGG) appears to have evolved later. Non-seed-making plants exclusively contain a glycine residue in P1, while a serine appears at this position for the first time with the emergence of gymnosperms (Figure 2A, P1). To our knowledge, no experimental data is available to document the transport properties of non-seed-making plant HKTs and whether or not this serine/glycine substitution translates into the same functional differences described for angiosperms. Monocotyledon sequences contain both configurations, while dicotyledons solely contain a serine residue. Sequence analysis reveals that the selectivity filter residue in P1 is part of a broader motif that evolved from TGL in non-seed-making plants to SSM in class I monocotyledons and dicotyledons. The highest variability in this motif is seen in class II monocotyledons, which may partially account for the high functional variety within this subgroup (Figure 2B, P1).

The functional importance of the residue at position 3 of the selectivity filter motif has been described in ion selectivity for AtHKT1;1 and TaHKT2;1, albeit weaker than the effect of mutations of the second position, which corresponds to the established selectivity filter residue [7]. It will be interesting to investigate whether substitutions in the first position residue of the motif result in any functional alterations.

### 2.4. Monocotyledons Are Sequence- and Function-Wise Versatile

Among angiosperms, monocotyledons demonstrate higher HKT sequence variability than dicotyledons. This conservation pattern is not surprising since monocotyledons express two different classes of HKT proteins, while only class I HKTs have been identified in dicotyledons so far [4]. Separating monocotyledons into class I and class II HKTs and early monocotyledons reveals sequence differences between classes that are often highly conserved (Figure 2B). In class II, the pore helix’s residues (approximate position indicated in Figure 2A) upstream of the selectivity filter residues are highly conserved. In contrast, in class I, these sequences show less conservation and are more diverse. This is exemplified by the class I residues D67, T73, and V75 (Y67, L73, and T75 in class II) in P1, and T699, F703, and T704 (L699, S703, and V704 in class II) in P2 (the numbering refers to the position in the multiple sequence alignment Figure S1 and Figure S2). In P3 of class I HKTs, on the other hand, only two positions in the helical structure are highly conserved (F861 and N865), while the pore domain helix in class II HKT is well conserved (NAxFMxV). In general, the pore domain’s helical region shows a higher grade of conservation in class II HKTs, compared to class I HKTs.

Also, the pore loops contain intriguing residues worth investigating, particularly around the selectivity filter. Similar to the SSM/TGL motif in P1 described above, amino acids in close proximity to the selectivity filter glycine residues are conserved (Figure 2). Some conserved residues are even class- or clade-specific. For example, in P3, a glutamate (E) is conserved next to the selectivity filter glycine only in seed-making plants. An asparagine (N) next to the same glutamate is highly conserved in class II HKTs of seed-making plants and, to some extent, in non-seed-making plants. Another class-specific conservation is a leucine (L) residue next to the selectivity glycine in P2 of class II HKT, which is conserved as phenylalanine (F) in class I HKTs. Mutation of this leucine to a phenylalanine in wheat HKT (TaHKT2;1-L247F) resulted in reduced Na^+^ currents and a reduced negative influence of high Na^+^ concentrations on K^+^ transport in yeast uptake experiments [2].

Two positions upstream of the selectivity filter glycine in P2 is an asparagine (N), which is conserved in monocotyledons and present in many, but not all, dicotyledons. Ali et al. mutated this residue in the *Arabidopsis thaliana* HKT1;1 to an aspartate (D) (AtHKT1;1-N211D), which is present in the dicotyledon halophyte *Thellungiella salsuginea* HKT1;2 (TsHKT1;2) at this position [44]. TsHKT1;2 is classified as a class I transporter due to a serine in the first pore loop, although it showed K^+^ uptake ability when expressed in yeast [10]. Interestingly, AtHKT1;1-N211D showed improved K^+^ transport relative to Na^+^, and transgenic *Arabidopsis* plants expressing AtHKT1;1-N211D demonstrated increased tolerance towards saline conditions. It is compelling that this position in monocotyledons contains a conserved asparagine, such as AtHKT1;1, independent of the class, and therefore, the functional properties (Na^+^ uniport vs. Na^+^-K^+^ symport).

The EVxSAYGNVG motif identified earlier in the pore domain of unit 4 is highly conserved in class I HKTs but only moderately conserved in the other two monocotyledon groups. Interestingly, an additional sequence motif (namely NxIF) appears to exist upstream of this motif and is only present in class II HKT sequences. The asparagine (N) in this motif is present in 74% of the HKT sequences examined and is conserved in dicotyledons and gymnosperms. The last amino acid of this motif, phenylalanine (F), is conserved in non-seed-making plants, gymnosperms, and class II HKTs, but diverts over time, becoming less frequent in class I HKTs and dicotyledons. Interestingly, this amino acid was mutated to leucine in a wheat class II channel (TaHKT2;1-F463L), as mentioned above [41]. The mutant showed not only an altered Na^+^ transport as observed for most HKT mutants described so far, but additionally had a reduced affinity for K^+^ in yeast uptake experiments, which is a known feature of class II HKTs.

### 2.5. Structure of the Second Transmembrane Segment in Unit 3

Plant HKT channels are homologues of bacterial and fungal Ktr and Trk channels [9,47]. The bacterial TrkH from *Vibrio parahaemolyticus* and KtrB from *Bacillus subtilis* have been functionally characterized to a great extent, and crystal structures are available for both proteins [48,49,50,51,52]. For the Trk/Ktr family, the presence and function of an internal loop in the center of the second transmembrane domain of unit 3 (U3M2) have been well-described and experimentally investigated [52]. This loop looms into the ion conduction pathway and can be pulled away from the pathway in response to conformational changes of regulatory proteins bound to Trk/Ktr on the intracellular side. Given these structural and conformational changes, it is hypothesized that this loop plays a role in channel gating [52,53].

**Figure 4:**
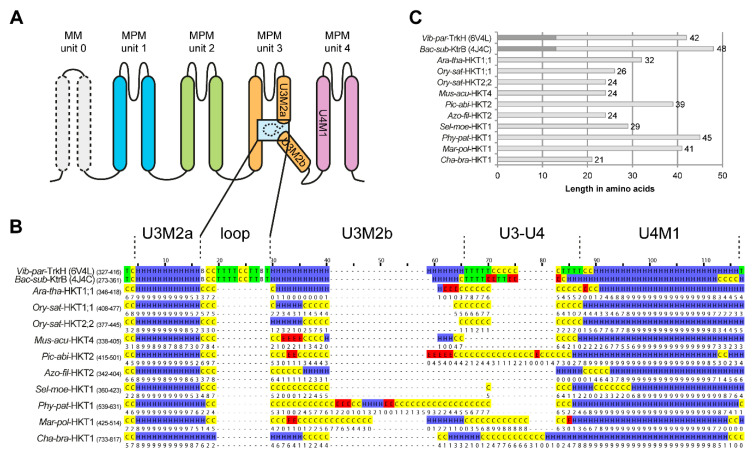
Secondary structure of the second transmembrane spanning segment of the third unit. Secondary structure analysis of the following sequences that represent each phylogenetic group. Sequences were aligned with MUSCLE [54]. Used sequences in this analysis: Ara-tha-HKT1;1 (*Arabidopsis thaliana*, AT4G10310), Ory-sat-HKT1;1 y Ory-sat-HKT2;2 (*Oryza sativa*, Os04g51820 y Q93XI5), Mus-acu-HKT4 (*Musa acuminate*, GSMUA_Achr1T23660_001), Pic-abi-HKT2 (*Picea abies*, MA_54668g0010), Azo-fil-HKT2 (*Azolla filiculoides*, Azfi_s0027.g023651), Sel-moe-HKT1 (*Selaginella moellendorffii*, PACid_15414191), Phy-pat-HKT1 (*Physcomitrella patens*, Pp3c1_15810V3), Mar-pol-HKT1 (*Marchantia polymorpha*, Mapoly0009s0076) and Cha-bra-HKT1 (*Chara braunii*, g19013). Additionally, sequences of *Vibrio parahaemolyticus* (Vib-par-TrkH) and *Bacillus subtilis* (Bac-sub-KtrB) are included for reference. (**A**) Schematic representation of the Trk/Ktr/HKT family protein structure. Structures in dashed lines (unit 0 and the internal loop in unit 3) are only present in some members of the family. Unit 0 is found in bacterial Trk channels only, and the internal loop is found in Trk and Ktr channels but not in plant HKT. (**B**) The secondary structure was determined with Porter5.0 that was recently tested among the best performing secondary prediction programs [55,56]. Prediction is based on three classes, which correspond to helix structures represented by H in blue (DSSP classes H, G, I), to strand structures represented by E in red (DSSP classes E, B), and to coil and turn structures represented by C in yellow (DSSP classes S, T.). Limits for transmembrane domains, loop and turn are approximated based on the secondary structure of TrkH and KtrB extracted from the respective crystal structures (TrkH: 6V4L, KtrB: 4J7C) using the visualization software VMD [57]. The secondary structure corresponds to the DSSP classes and colour code as described above with turns (T) coloured in green and isolated bridges (B) in white. Below each prediction is the confidence score for the prediction ranging from 0 (lowest confidence) to 9 (highest confidence). (**C**) Illustration of the sequence length between the end of the first half of the second transmembrane domain of unit 3 (U3M2a) and the beginning of the first transmembrane domain of unit 4 (U4M1). The length of the internal loop in TrkH and KtrB is marked in darker grey. Numbers next to the bars indicate the absolute length of sequence between the indicated transmembrane domains.

In HKT proteins, this loop structure has not attracted much interest so far as it is generally considered to be absent. Sequence alignment and secondary structure predictions of selected HKT sequences (one per evolutionary group) indicate that HKT sequences indeed do not align with the internal loop of KtrB and TrkH (Figure 4). The sequence lengths between the first half of the second transmembrane domain of unit 3 (U3M2a) and the beginning of the first transmembrane domain of unit 4 (U4M1) are also shorter in HKT proteins relative to those in KtrB and TrkH (except for *Mar-pol*-HKT1, *Phy-pat*-HKT1, and *Pic-abi*-HKT2) (Figure 4C).

Although these observations may suggest that the loop is absent in plant HKT proteins, the sequence between U3M2a and U4M1 is generally not predicted as a helix, or in the best of cases, the confidence for the helix prediction is low (Figure 4B). In general, the low prediction confidence in this region is independent of the secondary structure prediction. A low confidence value results from increased probabilities for more than one secondary structure. For instance, if a probability of 0.45 for coil, 0.4 for helix, and 0.15 for a sheet is calculated, the final prediction will be coil but with a low confidence value due to the also high prediction probability for the helix at this position (secondary structure prediction server Porter5.0, Figure 4B). Therefore, the predominantly low confidence in the secondary structure prediction gives an ambiguous view of the structural organization of U3M2b in plant HKT channels.

In conclusion, the secondary structure prediction does not reliably suggest that U3M2b forms a helix, nor that the internal loop is simply eliminated in plant HKT. Neither is U3M2b confidently predicted as a loop or coil. Instead, the structural organization of U3M2b in plant HKT channels appears ambiguous and deserves to be reconsidered and investigated.

## 3. HKT Gene Expression and Regulation

In addition to the functional diversity originating from structural variation, gene and protein regulation and localization of the transporter expression at particular developmental periods and specific plant tissues impart an additional layer of functional versatility. The following section summarizes the current literature regarding the regulation and localization of the expression of HKT family members in different plant species.

### 3.1. Gene Expression Regulation and Protein Localization

HKT channels are expressed in all plant parts from roots to shoots and leaves to the point of flowers (Table 1). They are often found in vascular tissue, mainly xylem parenchyma, but not exclusively. With one exception, all HKT proteins are localized to the plasma membrane. OsHKT1;3 is the only described family member so far that localizes to the Golgi membrane (Rosas-Santiago 2015). The proposed role of the Na^+^ selective OsHKT1;3 is the transport of Na^+^ into the cytoplasm, functioning as an alternative shunt conductance for proton pumps in the Golgi apparatus. HKT gene expression is often affected by stress conditions, such as high sodium or low potassium concentrations. However, there seems tob e no general pattern (Table 1). High Na+ concentration, for example, increases the gene expression of some HKT members, while it decreases the expression of others. Similarly, in some species, gene expression is upregulated in shoots and downregulated in roots, while the opposite effect is observed in other species. This shows that the expression and regulation of the HKT family are complex and equip plants with versatile mechanisms to react to stress situations.

### 3.2. Regulation of AtHKT1;1 Gene in Arabidopsis

While the function, structure, and evolutionary relationships of HKT proteins have been described, the molecular mechanisms regulating HKT expression are less well understood. Studies of *Arabidopsis*, rice, and other plant species have identified several transcription factors that modulate HKT gene expression (Table 2). The first transcription factor elucidated was AtbZIP24 (*Arabidopsis thaliana* basic leucine Zipper 24), which was shown to modulate *AtHKT1;1* (*A. thaliana* High-Affinity K^+^ Transporter 1;1) expression. *Arabidopsis* mutants with RNAi-mediated *AtbZIP24* repression showed an increase in *AtHKT1;1* transcript levels compared to wild-type *Arabidopsis*, indicating that AtbZIP24 functions as a negative regulator of *AtHKT1;1* expression [79]. Subsequent studies identified three additional *Arabidopsis* transcription factors—ARR1 (*Arabidopsis* Response Regulator 1), ARR12 (*Arabidopsis* Response Regulator 12), and ABI4 (Abscisic Acid Insensitive 4)—that modulate *AtHKT1;1* expression [80,81]. ARR1 and ARR12 were identified due to altered sodium accumulation phenotypes observed in *Arabidopsis* mutants of response regulators of the cytokinin signal transduction pathway. Additionally, *ARR1* and *ARR12* expression were rapidly induced by cytokinins. The expression of *AtHKT1;1* was decreased due to cytokinin treatment, while *AtHKT1;1* expression was increased in the *arr1 arr12* double mutant background. These observations indicated that ARR1 and ARR12 are negative regulators of *AtHKT1;1* and that cytokinins have a role in salt responses in *Arabidopsis* as cytokinins affected the expression of *ARR1*, *ARR12*, and *AtHKT1;1* [80].

The *Arabidopsis* transcription factor ABI4 and the abscisic acid signal transduction pathway are involved in HKT expression regulation. *Arabidopsis* abi4 mutants have increased salt tolerance due to higher *AtHKT1;1* expression and lower levels of sodium ion accumulation. Overexpressing *ABI4* resulted in salt hypersensitivity due to lower *AtHKT1;1* expression and a reduced accumulation of sodium ions. Additionally, ABI4 was shown to interact with the *AtHKT1;1* promoter through *in planta* chromatin immunoprecipitation and electrophoresis mobility shift assays. These results indicated that ABI4 is a negative regulator of *AtHKT1;1* expression and that abscisic acid is involved in salt responses in *Arabidopsis* [81].

### 3.3. Regulation of OsHKT Gene Expression in Rice

Multiple rice transcription factors, including OsMYBc (*O. sativa* MYBc), OsMYB106 (*O. sativa* MYB 106), and Osbhlh035 (*O. sativa* basic helix loop helix 035), have been reported to regulate *OsHKT* gene expression [65,83,84]. OsMYBc is a positive regulator of *OsHKT1;1* (*O. sativa High-affinity K^+^ Transporter 1;1*) expression. Yeast one-hybrid assays indicated that OsMYBc binds the *OsHKT1;1* promoter at specific conserved DNA segments (AAANATNC[C/T]). Knocking out OsMYBc decreased the salt-induced expression of *OsHKT1;1*, and introducing mutations into specific promoter segments decreased the promoter activity of *OsHKT1;1* [64].

OsMYB106 is another MYB transcription factor that also regulates an *OsHKT* gene. OsMYB106 interacts with OsBAG4 (*O. sativa* BCL-2-Associated Athanogene 4) and OsSUVH7 (*O. sativa* Suppressor of Variegation 3–9 Homolog 7) to form a complex that positively regulates *OsHKT1;5* (*O. sativa* High-affinity K^+^ Transporter 1;5) expression. The promoter of *OsHKT1;5* was shown to be methylated at specific DNA sequence patterns (CHG and CHH, with H being either A, T, or C), indicating that methylation plays a role in transcriptional regulation. OsSUVH7, which functions as a DNA methylation reader, was found to bind to the promoter of *OsHKT1;5* at CHG and CHH sites. OsBAG4, which functions as a chaperon regulator, was shown to bind OsSUVH7 directly. Finally, OsMYB106, OsBAG4, and OsSUVH7 formed a stable complex on the promoter of *OsHKT1;5*, increasing *OsHKT1;5* expression, indicating that the complex is a positive regulator of *OsHKT1;5* expression [83].

A final transcription factor that has been shown to positively modulate rice HKT gene expression is Osbhlh035, a modulator of *OsHKT1;3* (*O. sativa* High-affinity K^+^ Transporter 1;3) and *OsHKT1;5*. Rice *osbhlh035* mutants cannot recover from salt stress treatment and have an overaccumulation of sodium ions in their shoot tissue. Additionally, the expression of *OsHKT1;3* and *OsHKT1;5* is reduced in the *osbhlh035* mutant, compared to wild-type rice plants. These results indicated that Osbhlh035 positively regulates *OsHKT1;3* and *OsHKT1;5* gene expression [82].

### 3.4. Regulation of HKT Gene Expression in Other Plant Species

A poplar transcription factor, PalERF109 (*P. alba* Ethylene Response Factor 109), was identified as a regulator of *PalHKT1* (*P. alba* High-affinity K^+^ Transporter 1) expression. This transcription factor was identified due to its rapid increase in expression after salt treatment. Overexpression of *PalERF109* resulted in increased salt tolerance and increased *PalHKT1* expression. These results indicated that PalERF109 is a positive regulator of *PalHKT1* expression and that ethylene is involved in salt responses in poplar [84].

### 3.5. Non-Transcription Factor-Mediated HKT Gene Regulation

The barley transporter HvHKT2;1 (*H. vulgare* High-affinity K^+^ Transporter 2;1) was shown to have intron-retaining and exon-skipping variants in barley. These variants included *HvHKT2;1-e* (retained first exon region), *HvHKT2;1-i1* (retained first intron), and *HvHKT2;1-i2* (retained second intron). Salt treatments of barley resulted in a change in these variants’ ratios, with a drastic increase in *HvHKT2;1-i1* as salt stress increased. Additionally, the expression of *HvHKT2;1-i* (all introns retained) in the *trk1 trk2* yeast (*Saccharomyces cerevisiae*) strain defective in K^+^ uptake allowed for growth in media containing different concentrations of K^+^ ions. These results indicated that different intron-retaining and exon-skipping HKT variants play a role in salt responses in barley [85].

## 4. Conclusions

High functional variability of HKT channels and other transporters involved in the Na^+^ and K^+^ homeostasis demonstrates the complexity and diversity of Na^+^ detoxification and usage strategies. Further studies are necessary to fully decrypt different systems, especially their position and role within the cellular machinery. HKT proteins are regulated on many levels, including transcriptional and translational levels, and directly at the protein level. Therefore, it is essential to continue to study gene and protein regulation to fully understand the range of influence of saline stress- and K^+^ starvation-related effects and the role of HKT channels in these stresses.

Although the trend goes towards investigating and understanding gene expression regulation in response to environmental factors, the primary structure of HKT proteins still holds many interesting unexplored features worth investigating as they determine functionality. Some of these differences may account for or be related to the high functional diversity observed in this protein family, especially the class II subgroup. Deciphering novel structural and regulatory features will improve our understanding of how versatility is determined among HKT channels, which is crucial if we are to improve the salt tolerance of plants.

## Figures and Tables

**Table 1:** Gene expression and subcellular protein localization. Di—dicotyledons; Mono—monocotyledons; PM—plasma membrane.

Gene Name	Class	Species	Cotyledons	Gene Expression Localization	Expression Under Stress	Subcellular Protein Localization	Reference
*AtHKT1;1*	I	*Arabidopsis thaliana*	Di	Leaf and flower phloem, leaf xylem parenchyma; Root xylem parenchyma and phloem	Expression peaks at 30 mM K^+^ or Na^+^ in roots and shoots Lower or higher K^+^ and Na^+^ did not increase expression	PM	[23,26]
*CmHKT1;1*	I	* Cucurbita moschata *	Di	NA		PM	[58]
* McHKT1;1 *	I	*Mesembryanthemum crystallinum*	Di	Leaf, stems, flowers, and seedpods	High Na^+^: increased expression in leaves after 6 h, then decreased expression over 48 h	PM	[59]
*SlHKT1;1*	I	*Solanum lycopersicum*	Di	Vascular bundles of the main and secondary veins of leaves	High Na^+^: decreased expression in leave and stems but increased expression in roots	NA	[60]
*SlHKT1;2*	I	*Solanum lycopersicum*	Di	Vascular bundles of the main and secondary veins of leaf; Stellar cells of root	High Na^+^: decreased expression in leave and stems but increased expression in roots	NA	[60]
* VisHKT1;1 *	I	*Vitis vinifera*	Di	Root stele	NA	PM	[61]
* HvHKT1;5 *	*I*	*Hordeum vulgare*	Mono	Epidermis and parenchyma and pericycle cells adjacent to xylem vessels in the root stele	Low and high K^+^: increased root expression High Na^+^: increased expression in roots	PM	[62]
*OsHTK1;1*	I	*Oryza sativa*	Mono	Leaf bulliform cells and vascular tissues; Root epidermis, exodermis, cortex, and stele (mainly phloem)	Higher expression in shoots than in roots High Na^+^: increasing expression in roots but decreasing expression in shoots [63]	PM	[12,63,64,65]
*OsHTK1;3*	I	*Oryza sativa*	Mono	Leaf bulliform cells, vascular tissues, and mesophyll cells; Root cortex and stele vascular tissues	High Na^+^: increased expression in leaves and roots	Golgi membrane	[12,63,66,67]
*OsHTK1;4*	I	*Oryza sativa*	Mono	Leaf sheaths	High Na^+^: increased expression in leaves and roots	PM	[28,63,68]
*OsHTK1;5*	I	*Oryza sativa*	Mono	Parenchyma cells bordering xylem vessels in shoots and roots	High Na^+^: increased expression in leaves and roots	PM	[28,31,63,69]
*TaHKT1;4*	I	*Triticum aestivum*	Mono	Roots	NA	NA	[70]
*TaHKT1;5-D*	I	*Triticum aestivum*	Mono	Roots	NA	PM	[71]
*TmHKT1;4-A2*	I	*Triticum monococcum*	Mono	Leaf sheaths; Roots	NA	NA	[72]
*TmHKT1;5-A*	I	*Triticum monococcum*	Mono	Parenchyma and pericycle cells adjacent to xylem vessels in the root stele	NaCl treatment did not affect expression in roots	NA	[71,73]
*HvHKT2;1*	*II*	*Hordeum vulgare*	Mono	Leaf blade and sheath; Root cortex cells	Low K^+^: increased expression in leaf sheaths, leaf blades, and roots High Na^+^: decreased expression in leaf sheaths and roots but increased expression in leaf blades	NA	[34]
*OsHKT2;1*	II	*Oryza sativa*	Mono	Leaf bulliform cells, vascular tissues, and mesophyll cells; Root epidermis, endodermis, exodermis, cortex, and stele (mainly phloem)	Low K^+^: increased expression in roots High K^+^ or Na^+^: decreased expression in roots	PM	[12,19,33]
*OsHKT2;2*	II	*Oryza sativa*	Mono	Roots	Low K^+^ and/or low Na^+^: increased expression High K^+^ and/or high Na^+^: decreased expression	NA	[19]
* OsHKT2;3 *	II	*Oryza sativa*	Mono	Leaf blade and sheath; Roots	NA	NA	[74]
* OsHKT2;4 *	II	*Oryza sativa*	Mono	Leaf blade and sheath, leaf epidermal cells, vasculature of spikelets, leaves, and stems; Root vasculature	NA	PM	[13,17,75]
*SvHKT2;1*	II	*Sporobolus virginicus*	Mono	NA	NA	PM	[75]
*SvHKT2;2*	II	*Sporobolus virginicus*	Mono	NA	NA	PM	[75]
*TaHKT2;1*	II	*Triticum aestivum*	Mono	Vasculature tissue of leaf mesophyll; Root cortical cells	NA	NA	[1,76]
* TaHKT2;3 *	II	*Triticum aestivum*	Mono	Shoots; Roots	NA	NA	[76,77]
*ZmHKT2*	II	*Zea mays*	Mono	Roots, with highest expression in the stele	NA	PM	[78]

**Table 2:** Transcription factors that modulate *HKT* expression in plants.

Species	Transcription Factor	Regulator	HKT	Reference
*Arabidopsis thaliana*	ABI4	Negative	*AtHKT1;1*	[81]
	ARR1	Negative	*AtHKT1;1*	[80]
	ARR12	Negative	*AtHKT1;1*	[80]
	AtbZIP24	Negative	*AtHKT1;1*	[79]
*Oryza sativa*	Osbhlh035	Positive	*OsHKT1;3* and *OsHKT1;5*	[82]
	OsMYB106 (with OsBAG4 and OsSUVH7)	Positive	*OsHKT1;5*	[83]
	OsMYBc	Positive	*OsHKT1;1*	[64]
*Populus alba*	PalERF109	Positive	*PalHKT1;2*	[84]

## Supplementary Materials

Supplementary materials can be found at https://www.mdpi.com/1422-0067/22/4/1892/s1. References are included here in the reference list [4,5,6,86,87].

## Abbreviations

HKT	High-affinity K^+^ (potassium) Transporters
Na^+^	Sodium
K^+^	Potassium
