# Supplementary material for: Plant HKT Channels: An Updated View on Structure, Function and Gene Regulation
Source: Int J Mol Sci. 2021 Feb 14;22(4):1892. doi: 10.3390/ijms22041892 (PMC7918770; doi:10.3390/ijms22041892)
Supplement: Supplementary file 1 [file ijms-22-01892-s001.pdf]

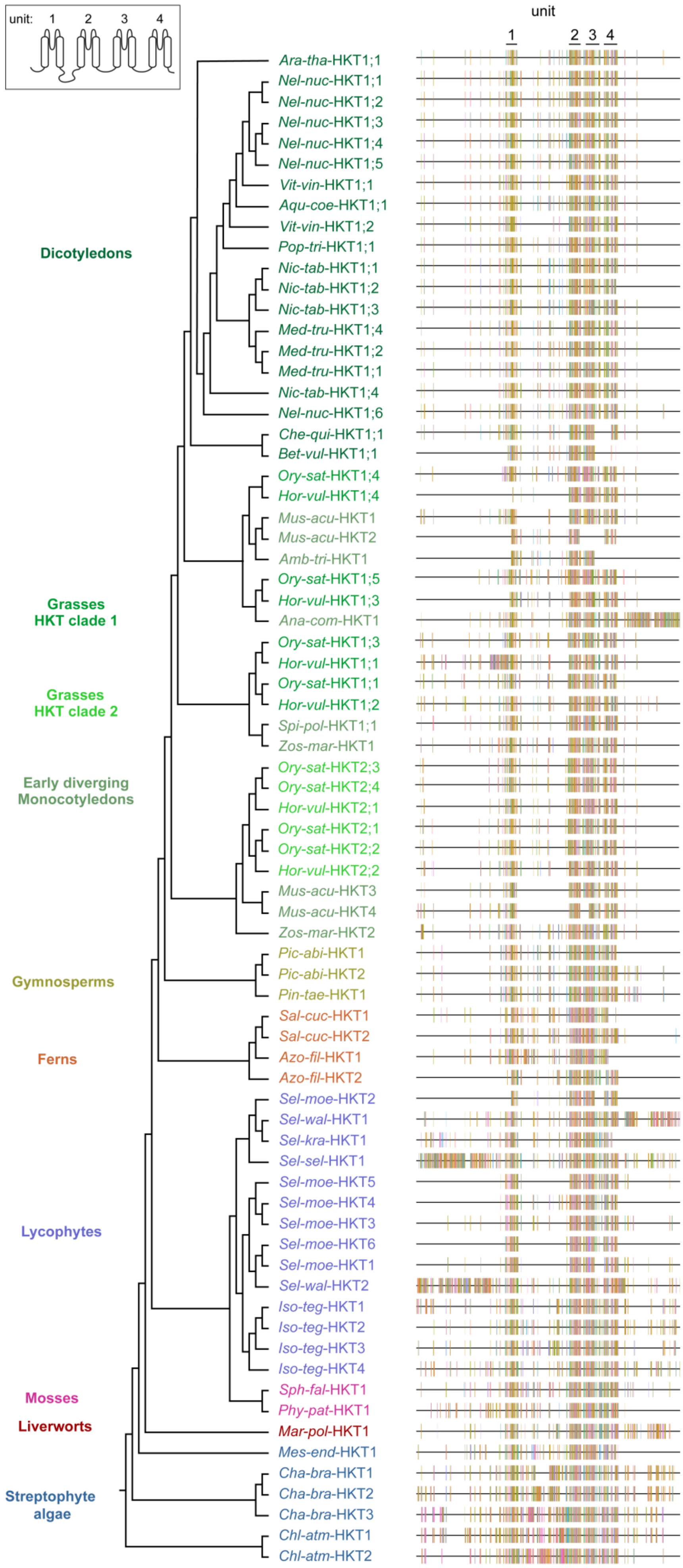

Figure 1S. Phylogenetic relationship of plant HKT channels. Sequences were obtained and the phylogenetic tree was generated according to Dreyer and colleagues, using the below mentioned resources and focusing in particular on the transmembrane regions [61]. BLAST searches were performed based on HKT sequences of *Arabidopsis thaliana*, *Sphagnum fallax, Marchantia polymorpha, Populus trichocarpa,* and *Selaginella moellendorfi* against genes of other plant organisms with minimum query coverage of 50% and an E-value less or equal of 0.01 as cut-offs. DNA sequences were translated into protein sequences with an in-house Perl script. Multiple sequence alignment was then performed with MAFFT v7.471 [62]. The tree was reconciled with the evolutionary plant species tree. The simplified sequence alignment on the right indicates the positions of the four transmembrane-pore-transmembrane modules (unit 1-4). Sequences used in this analysis: dicotyledons: *Arabidopsis thaliana*: *Ara-tha*-HKT1;1 (AT4G10310); *Nelumbo nucifera*: *Nel-nuc*-HKT1;1 (XP_010263413), *Nel-nuc*-HKT1;2 (XP_010263423), *Nel-nuc*-HKT1;3 (XP_010263267), *Nel-nuc*-HKT1;4 (XP_010263268), *Nel-nuc*-HKT1;5 (XP_010263271), *Nel-nuc*-HKT1;6 (XP_010263272); *Vitis vinifera*: *Vit-vin*-HKT1;1 (CBI40144), *Vit-vin*-HKT1;2 (CBI40134); *Aquilegia coerulea*: *Aqu-coe*-HKT1;1 (PIA43899); *Populus trichocarpa*: *Pop-tri*-HKT1;1 (XP_002325229); *Nicotiana tabacum*: *Nic-tab*-HKT1;1 (XP_016481033), *Nic-tab*-HKT1;2 (XP_009789270), *Nic-tab*-HKT1;3 (XP_016464629), *Nic-tab*-HKT1;4 (XP_016459984); *Medicago truncatula*: *Med-tru*-HKT1;1 (Medtr6g092820), *Med-tru*-HKT1;2 (Medtr6g092840), *Med-tru*-HKT1;4 (Medtr6g092940); *Chenopodium quinoa*: *Che-qui*-HKT1;1 (XP_021774589); *Beta vulgaris*: *Bet-vul*-HKT1;1 (XP_010690257); monocotyledons: *Oryza sativa*: *Ory-sat*-HKT1;1 (Os04g51820), *Ory-sat*-HKT1;3 (Os02g07830), *Ory-sat*-HKT1;4 (Os04g51830), *Ory-sat*-HKT1;5 (Os01g20160), *Ory-sat*-HKT2;1 (Os06g48810), *Ory-sat*-HKT2;2 (Q93XI5), *Ory-sat*-HKT2;3 (Os01g34850), *Ory-sat*-HKT2;4 (Os06g48800); *Hordeum vulgare*: *Hor-vul*-HKT1;1 (KAE8801129), *Hor-vul*-HKT1;2 (KAE8777434), *Hor-vul*-HKT1;3 (KAE8777465), *Hor-vul*-HKT1;4 (KAE8777433), *Hor-vul*-HKT2;1 (KAE8806779), *Hor-vul*-HKT2;2 (KAE8806777); *Spirodela polyrhiza*: *Spi-pol*-HKT1;1 (Spipo10G0003400); *Amborella trichopoda*: *Amb-tri*-HKT1 (AmTr_v1.0_scaffold00025.231); *Musa acuminate*: *Mus-acu*-HKT1 (GSMUA_Achr6T30960_001), *Mus-acu*-HKT2 (GSMUA_Achr7T16030_001), *Mus-acu*-HKT3 (GSMUA_Achr1T23670_001), *Mus-acu*-HKT4 (GSMUA_Achr1T23660_001); *Zostera marina*: *Zos-mar*-HKT1 (KMZ58913), *Zos-mar*-HKT2 (KMZ75022); *Ananas comosus*: *Ana-com*-HKT1 (Aco008305); gymnosperms: *Picea abies*: *Pic-abi*-HKT1 (MA_10435813g0010), *Pic-abi*-HKT2 (MA_54668g0010); *Pinus taeda*: *Pin-tae*-HKT1 (PITA_00520); ferns: *Salvinia cucullata*: *Sal-cuc*-HKT1 (Sacu_v1.1_s0005.g002887), *Sal-cuc*-HKT2 (Sacu_v1.1_s0036.g011647); *Azolla filiculoides*: *Azo-fil*-HKT1 (Azfi_s0004.g008508), *Azo-fil*-HKT2 (Azfi_s0027.g023651); lycophytes: *Selaginella moellendorffii*: *Sel-moe*-HKT1 (PACid_15414191), *Sel-moe*-HKT2 (PACid_15414777), *Sel-moe*-HKT3 (PACid_15422070), *Sel-moe*-HKT4 (PACid_15420572), *Sel-moe*-HKT5 (PACid_15412354), *Sel-moe*-HKT6 (PACid_15412619); *Selaginella wallacei*: *Sel-wal*_HKT1 (JKAA-2005360), *Sel-wal*_HKT2 (JKAA-2004415); *Selaginella kraussiana*: *Sel-kra*-HKT1 (ZFGK-2041766); *Selaginella selaginoides*: *Sel-sel*-HKT1 (KUXM-2007374); *Isoetes tegetiformans*: *Iso-teg*-HKT1 (PKOX-2007255), *Iso-teg*-HKT2 (PKOX-2013827), *Iso-teg*-HKT3 (PKOX-2100389), *Iso-teg*-HKT4 (PKOX-2010020); mosses: *Sphagnum fallax*: *Sph-fal*-HKT1 (Sphfalx0141s0038); *Physcomitrella patens*: *Phy-pat*-HKT1 (Pp3c1_15810V3); liverworts: *Marchantia polymorpha*: *Mar-pol*-HKT1 (Mapoly0009s0076); streptophyte algae: *Mesotaenium endlicherianum*: *Mes-end*-HKT1 (ME000348S06055); *Chlorokybus atmophyticus*: *Chl-atm*-HKT1 (Chrsp24S03841), *Chl-atm*-HKT2 (Chrsp24S03855); *Chara braunii*: *Cha-bra*-HKT1 (g19013), *Cha-bra*-HKT2 (g23076), *Cha-bra*-HKT3 (g48299). Sequences were numbered following recommendations of Platten and colleagues for dicotyledons and monocotyledons [4]. Evolutionary older sequences were numbered consecutively as the rules mentioned before apply for the two-clade system of land plants and are not straight forward applicable for evolutionary older sequences, as pointed out by Haro and colleagues [5,6].

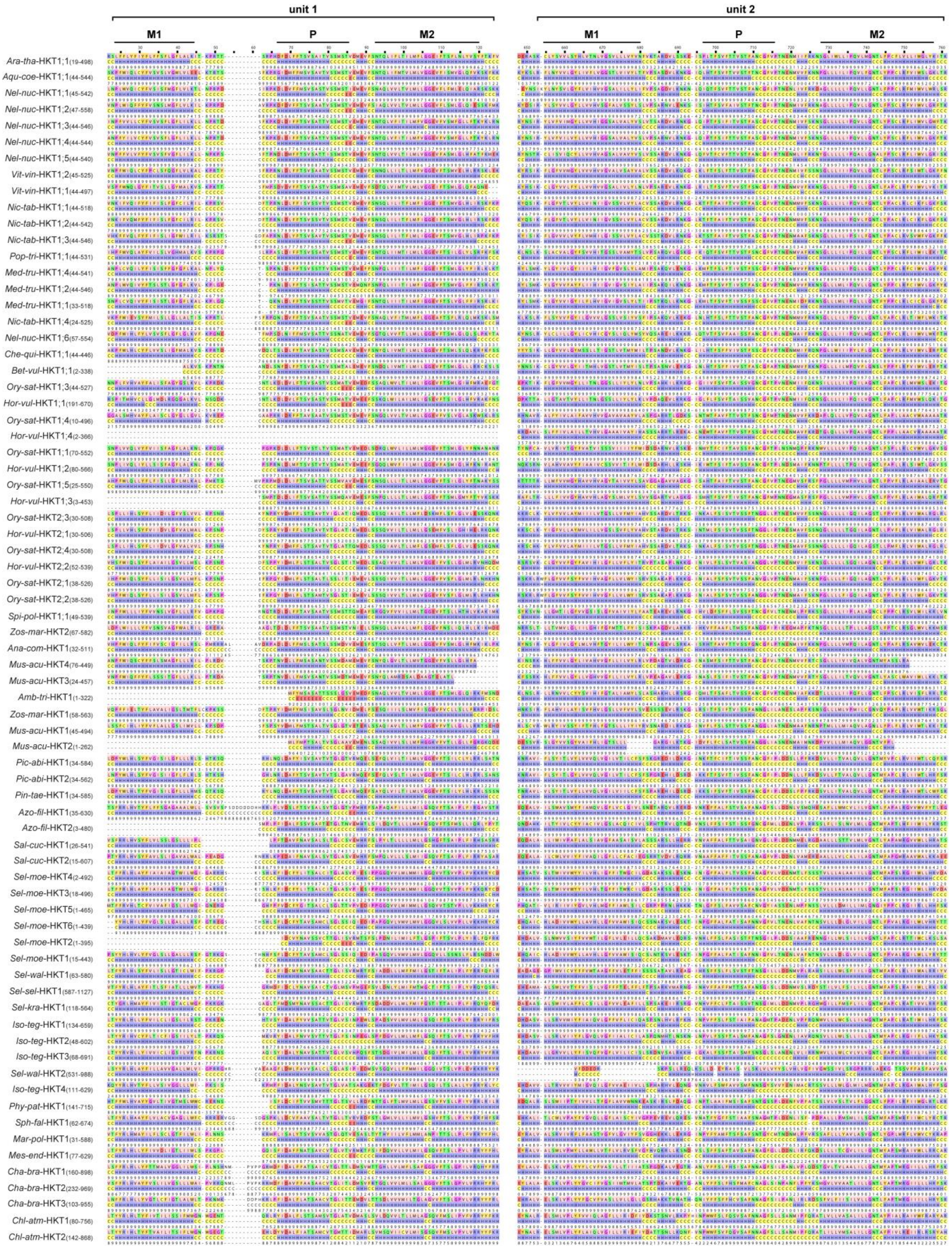

Figure S2. Multiple sequence alignment including secondary structure prediction of 73 HKT channel sequences. Multiple sequence alignment performed with MAFFT of HKT sequences used for the analyses described in the main text. Only the four units including first transmembrane domain (M1), pore domain (P) and second transmembrane domain (M2) are represented. In unit 3, the second part of transmembrane domain 2 is named (M/C)2b because the secondary structure is not assured. Three lines are associated with each HKT protein. (1) The primary amino acid sequence, (2) the secondary structure prediction, and (3) the confidence of the secondary structure prediction. The secondary structure was determined with Porter5.0 that was recently tested among the best performing secondary prediction programs (Torrisi, Kaleel, and Pollastri 2019; Musci, Maruccia, and Ferretti 2019). Three classes are predicted, which correspond to helix structures H in blue (DSSP classes H, G, I), to strand structures E in red (DSSP classes E, B), and to coil and turn structures C in yellow (DSSP classes S, T, .).

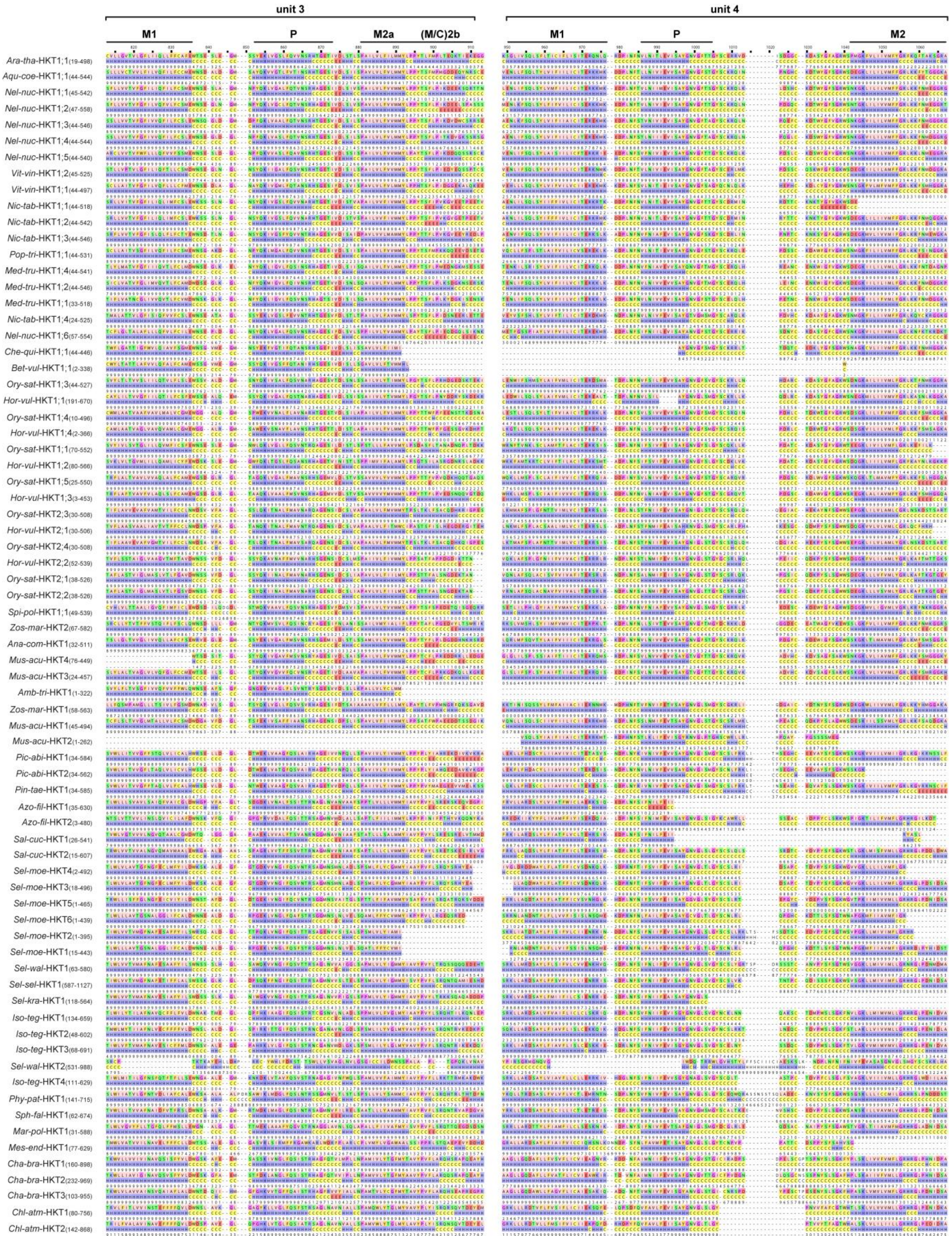
unit 3
unit 4
M1
P
M2a
(M/C)2b
M1
P
M2
Ara-tha-HKT1;1(19-498)
Aqu-coe-HKT1;1(44-544)
Nel-nuc-HKT1;1(45-542)
Nel-nuc-HKT1;2(47-558)
Nel-nuc-HKT1;3(44-546)
Nel-nuc-HKT1;4(44-544)
Nel-nuc-HKT1;5(44-540)
Vit-vin-HKT1;2(45-525)
Vit-vin-HKT1;1(44-497)
Nic-tab-HKT1;1(44-518)
Nic-tab-HKT1;2(44-542)
Nic-tab-HKT1;3(44-546)
Pop-tri-HKT1;1(44-531)
Med-tru-HKT1;4(44-541)
Med-tru-HKT1;2(44-546)
Med-tru-HKT1;1(33-518)
Nic-tab-HKT1;4(24-525)
Nel-nuc-HKT1;6(57-554)
Che-qui-HKT1;1(44-446)
Bet-vul-HKT1;1(2-338)
Ory-sat-HKT1;3(44-527)
Hor-vul-HKT1;1(191-670)
Ory-sat-HKT1;4(10-496)
Hor-vul-HKT1;4(2-366)
Ory-sat-HKT1;1(70-552)
Hor-vul-HKT1;2(80-566)
Ory-sat-HKT1;5(25-550)
Hor-vul-HKT1;3(3-453)
Ory-sat-HKT2;3(30-508)
Hor-vul-HKT2;1(30-506)
Ory-sat-HKT2;4(30-508)
Hor-vul-HKT2;2(52-539)
Ory-sat-HKT2;1(38-526)
Ory-sat-HKT2;2(38-526)
Spi-pol-HKT1;1(49-539)
Zos-mar-HKT2(67-582)
Ana-com-HKT1(32-511)
Mus-acu-HKT4(76-449)
Mus-acu-HKT3(24-457)
Amb-tri-HKT1(1-322)
Zos-mar-HKT1(58-563)
Mus-acu-HKT1(45-494)
Mus-acu-HKT2(1-262)
Pic-abi-HKT1(34-584)
Pic-abi-HKT2(34-562)
Pin-tae-HKT1(34-585)
Azo-fil-HKT1(35-630)
Azo-fil-HKT2(3-480)
Sal-cuc-HKT1(26-541)
Sal-cuc-HKT2(15-607)
Sel-moe-HKT4(2-492)
Sel-moe-HKT3(18-496)
Sel-moe-HKT5(1-465)
Sel-moe-HKT6(1-439)
Sel-moe-HKT2(1-395)
Sel-moe-HKT1(15-443)
Sel-wal-HKT1(63-580)
Sel-sel-HKT1(587-1127)
Sel-kra-HKT1(118-564)
Iso-teg-HKT1(134-659)
Iso-teg-HKT2(48-602)
Iso-teg-HKT3(68-691)
Sel-wal-HKT2(531-988)
Iso-teg-HKT4(111-629)
Phy-pat-HKT1(141-715)
Sph-fal-HKT1(62-674)
Mar-pol-HKT1(31-588)
Mes-end-HKT1(77-629)
Cha-bra-HKT1(160-898)
Cha-bra-HKT2(232-969)
Cha-bra-HKT3(103-955)
Chl-atm-HKT1(80-756)
Chl-atm-HKT2(142-868)
